# The cost-effectiveness of growth hormone replacement therapy (Genotropin®) in hypopituitary adults in Sweden

**DOI:** 10.1186/1478-7547-11-24

**Published:** 2013-09-30

**Authors:** Kristian Bolin, Rickard Sandin, Maria Koltowska-Häggström, Jane Loftus, Christin Prütz, Björn Jonsson

**Affiliations:** 1 Department of Economics, Centre for Health Economics, University of Gothenburg, P.O. Box 640, SE-405 30 Gothenburg Sweden; 2 Pfizer AB, Sollentuna, Stockholm, Sweden; 3 Pfizer, Endocrine Care, Pfizer Inc, Sollentuna, Sweden; 4 Pfizer Ltd, Walton Oaks, UK; 5 Department of Women’s and Children’s Health, Uppsala University, Uppsala, Sweden

**Keywords:** Cost effectiveness, Adults, Growth hormone, QALY

## Abstract

**Background:**

To evaluate the cost-effectiveness of growth hormone (GH) treatment (Genotropin®) compared with no GH treatment in adults with GH deficiency in a Swedish societal setting.

**Methods:**

A Markov-type cost-utility simulation model was constructed and used to simulate, for men and women, morbidity and mortality for GH-treated and -untreated individuals over a 20-year period. The calculations were performed using current available prices concerning morbidity-related healthcare costs and costs for Genotropin®. All costs and treatment effects were discounted at 3%. Costs were expressed in Euro (1€ = 9.03 SEK). GH-treated Swedish patients (*n* = 434) were identified from the KIMS database (Pfizer International Metabolic Database) and untreated patients (*n* = 2135) from the Swedish Cancer Registry and the Hospital Discharge Registry.

**Results:**

The results are reported as incremental cost per quality-adjusted life year (QALY) gained, including both direct and indirect costs for GH-treated *versus* untreated patients. The weighted sum of all subgroup incremental cost per QALY was €15,975 and €20,241 for men and women, respectively. Including indirect cost resulted in lower cost per QALY gained: €11,173 and €10,753 for men and women, respectively. Key drivers of the results were improvement in quality of life, increased survival, and intervention cost.

**Conclusions:**

The incremental cost per QALY gained is moderate when compared with informal thresholds applied in Sweden. The simulations suggest that GH-treatment is cost-effective for both men and women at the €55,371 (SEK 500,000 – the informal Swedish cost-effectiveness threshold) per QALY threshold.

## Background

Growth hormone deficiency (GHD) in adults with hypopituitarism can persist from childhood or can be acquired in adulthood, most often as a result of pituitary or peripituitary tumours or their treatment. The Society for Endocrinology (UK) estimates the prevalence of GHD in the adult population to be three in 10,000 [1], which equates to approximately 2700 adults in Sweden.

Adults suffering from GHD are typically characterised by impaired health-related quality of life (QoL) along with increased risks of cardio- and cerebrovascular morbidity [2] and mortality [3,4]. The most affected QoL domains are energy levels, vitality, life-drive, emotional functioning, social isolation, and cognitive abilities such as memory [5]). A study based on data collected prior to the introduction of growth hormone treatment (GHT) showed that additional healthcare costs resulting from GHD in Sweden were estimated to be SEK 36,000 (approximately €3,987) per patient and year [6]. Patients with untreated GHD consumed more healthcare resources (doctor visits and hospital stays) compared with the general population. Patients with GHD were also more frequently absent from work due to sickness and more likely to claim disability pension compared with the general population [6,7]. Similar results have been found for other countries [8,9].

Growth hormone therapy (GHT) in adults with GHD has been shown to improve QoL [10-12] and to reduce cardio- and cerebrovascular morbidity [13,14]. In addition, there is indirect evidence suggesting that GHT also reduces mortality [15]. The increases in drug cost associated with the introduction of GHT have therefore been offset by a reduction in healthcare utilisation and less sickness absenteeism [16,17].

Cost-effectiveness analysis of health technologies are becoming a pre-requisite for healthcare decision-making in many countries. The peer-reviewed literature addressing the cost-effectiveness of GHT in adults is lacking [18,19] and limited to only one publication in the past decade, based on an analysis of the cost-effectiveness of GH on behalf of the National Institute for Clinical Excellence (NICE) in UK [19]. The objective of this paper was to evaluate the cost-effectiveness of GHT compared with no GHT in adults suffering from GHD, using Swedish data, based on treatment effects, morbidity, mortality and costs. As the present study was performed from the societal perspective, both direct and indirect costs were taken into consideration. We calculated the cost-effectiveness of GHT by including cardiovascular morbidity and mortality assessments as well as novel methods to evaluate economic impact of QoL data.

## Methods

In this section we will specify the methods that have been used for the analysis. At the end of the section a shortlist of assumptions is provided.

### Subjects

Patients in the GHT group were identified from the KIMS (Pfizer International Metabolic Database) [20]—434 of the 1459 Swedish patients identified from KIMS met the following inclusion criteria and were included in the analysis: (1) Resided in Sweden; (2) GHD due to non-functioning pituitary adenoma (NFPA); (3) not receiving GHT for ≥6 months prior to entry into the database; (4) no missing QoL values at baseline; and (5) aged >18 years at entry into KIMS. The study period was from 1995–2011 and the GHT prescribed was Genotropin®.

In the untreated group, patients diagnosed with pituitary adenoma were identified through the Swedish Cancer Registry and the Hospital Discharge Registry administered by The National Board of Health and Welfare. Patients (*n* = 2135) aged 18–75 years were included in the analysis; the study period was from 1987–1992. Eighty percent of patients had undergone surgical removal of the macroadenoma, therefore it was assumed that the majority of patients suffered from macroadenomas, which are associated with a high incidence of concomitant pituitary failure [21]. Patients with acromegaly, Cushing’s disease, or malignant pituitary adenoma, and patients who had died within 1 month after diagnosis of pituitary adenoma were excluded. Permission to use the registry data on pituitary tumours in Sweden was granted by the ethics committee of the Karolinska Institute.

### Simulation model

A simulation model was constructed to assess the cost-effectiveness of GHT in patients with GHD due to NFPA. The model is a Markov-type simulation model that tracks a cohort of patients over 20 years. Each year (cycle) the population is at risk of moving into specific states of illness or death. The proportion of patients that moves from one state to another was calculated on given age- and gender-specific cardio- and cerebrovascular morbidity and mortality rates. The model performs simultaneous but separate calculations for a population that received GHT and a population that did not receive GHT. (See Additional file 1 for more details on the calculation).

**Figure 1:**
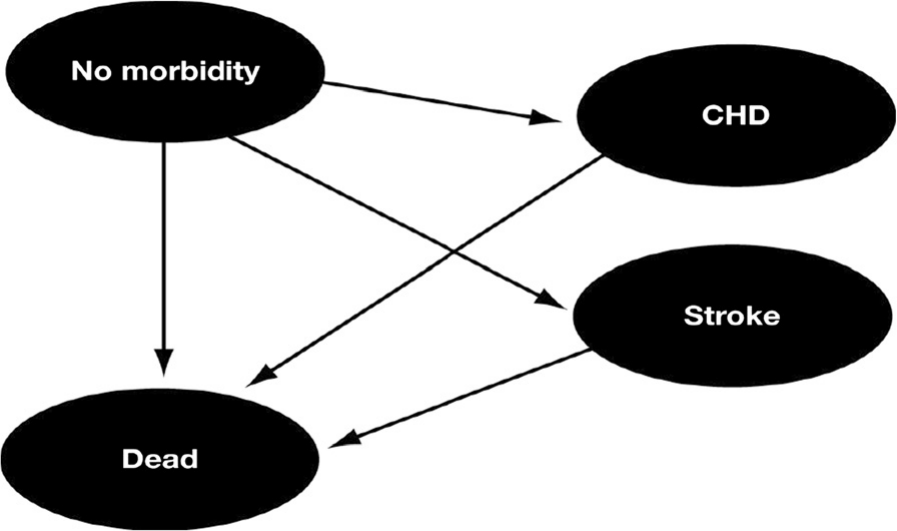
**Markov structure for cost**-**effectiveness model.** Possible transitions are indicated by arrows. Diseases considered: CHD, coronary heart disease.

The model distinguishes between six health states, defined as follows: (a) no morbidity; (b) new coronary heart disease (CHD) event; (c) previous CHD event; (d) new stroke; (e) previous stroke; and (f) dead (Figure 1). The model does not allow for transition from one morbidity state to any other state except death; neither does it allow for more than one event per cycle. However, it is possible to have a new CHD or stroke event, respectively. The model distinguishes between different patient groups based on: (1) gender; (2) age (18–30 yr, 31–54 yr, 55–65 yr, and ≥66 yr); and (3) QoL–Assessment of Growth Hormone Deficiency in Adults (QoL-AGHDA) (<2, 2–6, 7–11, 12+) score at baseline, resulting in calculations being performed for 32 different patient groups, and six health states.

### Data used in the model

The simulation model was provided with the following input: (1) cerebro- and cardiovascular morbidity and mortality risks for GHT and untreated patients; (2) QoL as measured by the QoL-AGHDA before the start of GHT (at baseline) and after 1 year of GHT; (3) average annual direct healthcare costs caused by disease (CHD and stroke); (4) intervention costs (drug dosage × Genotropin® unit cost); (5) cost of sick leave; and (6) average net contribution to society (production minus consumption).

### Cerebro- and cardiovascular morbidity and mortality risks

Morbidity and mortality data was obtained from the Swedish hospital discharge and causes of death registries, respectively, for the untreated patients and from the KIMS database for the GHT patients. Relative morbidity and mortality risks were calculated according to age and gender groups (Table 1). A further division of risks based on QoL was not possible because there were no QoL data for the untreated group. Therefore, the number of individuals in each state and at each point in time does not differ according to QoL in the model. Mean age was 54 years and 53 years in the GHT and untreated groups, respectively, but the difference was not statistically significant. Mean time of observation was higher in the GHT group (6 yr) as compared with the untreated group (5.5 yr) and the difference was significant (*P* < 0.003). Each group had a similar proportion of men and women (54% and 51% male for the treated and untreated groups, respectively).

**Table 1:** Morbidity risk of CHD and stroke and mortality risk (treated and untreated adults with GHD)

	**Yearly morbidity risk**				**Yearly mortality risk**	
	**CHD**		**Stroke**		**Mortality**	
	**Treated**	**Untreated**	**Treated**	**Untreated**	**Treated**	**Untreated**
Men, years						
18–30	0	0	0	0	0	0.0145
31–54	0.0044	0.0045	0.0044	0.0038	0.0054	0.0179
55–65	0.0118	0.0281	0.0074	0.0144	0.0103	0.0406
65+	0.0185	0.0408	0.0123	0.0198	0.0559	0.0906
Women, years						
18–30	0	0	0	0	0	0.0039
31–54	0.0022	0.0055	0.0011	0.0025	0.0045	0.0122
55–65	0.0045	0.0145	0.0067	0.0087	0.00159	0.0378
65+	0.0046	0.0306	0.0183	0.0164	0.0285	0.0673

CHD, coronary heart disease; GHD, growth hormone deficiency.

Sources of data: Swedish hospital discharge and causes of death registries (untreated); KIMS database (treated).

### Quality of life as measured by QoL-AGHDA

Prior to initiation of GHT, patients in the KIMS database completed the QoL-AGHDA questionnaire [22], which is a disease-specific measure of QoL for adults with GHD. A validated questionnaire with good reliability and a high level of internal consistency [23], QoL-AGHDA consists of 25 items that evoke yes/no (=1/0) answers to specific problems; the sum of yes (=1) answers constitutes the QoL-AGHDA score. A high QoL-AGHDA score denotes a poor QoL. A Swedish version of the QoL-AGHDA was produced simultaneously using dual translation panels during the measure development. Although the QoL-AGDHA questionnaire does not provide preference-based assessments of individual health applicable for economic decision-making, the QoL-AGDHA score was translated into EQ-5D scores, a generic measure of QoL, (EuroQoL Group, Rotterdam, The Netherlands) by applying an algorithm recently derived specifically for the Swedish population (see Additional file 1 for more detail) [24]. Average QoL-AGDHA scores at baseline and average treatment effects (reduction in the QoL-AGDHA score) for each subgroup were calculated based on the KIMS patient group. For the case scenario at baseline, it was assumed that the treatment effect (reduction in QoL-AGDHA score) resulting from GHT appeared within the first year of treatment. For subsequent years (years 2–20), utility-score changes were induced by advancing age. The comparative population that did not receive GHT was assumed to have the same baseline QoL-AGDHA scores as the treated population, but no first-year treatment effect.

### Average annual direct healthcare costs caused by disease (CHD and stroke)

Direct cost estimates were morbidity-related healthcare cost data that included both inpatient and outpatient care. The data were collected from a county in southern Sweden (Region of Skåne) that is considered demographically representative of the Swedish population. Healthcare costs during the first year of disease were distinguished from those costs incurred in the subsequent years after diagnosis and as a result of the disease. The cost estimates were based on Swedish individual diagnosis-related data on all inpatient stays and outpatient visits [25], which were inflated to 2011 year price levels (Consumer Price Index, Statistics Sweden, http://www.scb.se). The healthcare consumption of all individuals diagnosed with CHD or stroke was followed during a 3-year period. The average cost during the first year following initial diagnosis was used as an estimate of the first-year healthcare cost: €5,331 (SEK 48,135) and €8,729 (SEK 78,824) for CHD and stroke, respectively; the average cost during the second and third years was used as an estimate of the healthcare costs during subsequent years: €1,528 (SEK 13,799) and €2,095 (SEK 18,915) for CHD and stroke, respectively. Cost of physician (specialist) visits was €373 (SEK 3,367).

### Intervention costs

The intervention cost is the cost of GHT (Genotropin®), i.e. the amount of utilisation (dose) × the unit cost €31 (SEK 282) per milligram (the Swedish Dental and Pharmaceutical Benefits Agency). Information of the mean consumption of Genotropin® per year for each of the age, gender and QoL-AGDHA (baseline) groups was provided from the study population in the KIMS database (Table 2).

**Table 2:** Estimated age-specific QoL-AGHDA score at baseline, first-year treatment effect, and drug utilisation

**QoL-AGHDA score by age, years**	**Men**		**Women**		**Drug dosage(mg/day)**	
	**Baseline**	**1st-year treatment**	**Baseline**	**1st-year treatment**	**Men**	**Women**
12+						
18–30	16.37	−7.05^a^	14.60	−5.48	0.30	0.22
31–54	16.25	−6.53	16.57	−5.48	0.26	0.36
55–65	16.06	−9.40	17.13	−4.25	0.23	0.29
65+	16.80	−9.25	14.20	−2.00	0.39	0.25
7–11						
18–30	8.92	−3.43	8.67	−2.33	0.24	0.25
31–54	8.37	−3.43	8.64	−2.65	0.23	0.33
55–65	9.25	−4.75	8.21	−2.44	0.23	0.28
65+	9.15	−3.54	9.17	−4.42	0.25	0.21
2–6						
18–30	3.20	1.00	4.00	0	0.54	0.18
31–54	3.85	−1.10	4.17	−1.81	0.30	0.44
55–65	4.07	−1.71	3.92	−1.60	0.28	0.25
65+	4.2	0	4.14	−1.67	0.24	0.25
<2						
18–30	0.50	3.00	1.00	−1.00	0.41	0.26
31–54	0.22	0.60	0.40	0	0.34	0.43
55–65	0.40	0.81	0.14	0	0.28	0.30
65+	0.08	1.44	0.67	0.5	0.18	0.23

^a^There was no observation in this group.

Qol-AGHDA, Quality-of-Life Adult Growth Hormone Deficiency Assessment.

Sources of data: KIMS database.

### Cost of illness-related absenteeism

The simulation model takes into account two different sources of indirect costs: changes in illness-related absenteeism and cost resulting from indirect effects due to difference in mortality. Evaluations are based on the human capital approach [26]. Information concerning reduction in illness-related absenteeism was taken from Jonsson *et al*. [7], where sick leave and early pension was studied in a Swedish population for the year 1989. Patients included in the National Cancer Registry who were diagnosed before the end of 1989, alive in 1989, and aged 16–64 years, were selected for the study. A total of 809 individuals remained eligible for analysis and the Swedish National Social Insurance Board was able to supply data on sick leave and disability pension status for 803 and 802 of these patients, respectively. The average number of reimbursed sick days was 40.2 among the untreated patients and 24.0 in the control group, resulting in a gain of 16 workdays for GHT patients. The gain was assumed to be the same between all groups below the age of 65 years, and over time. The value of the production resulting from a workday was estimated using average labour income in Sweden 2011: €182 (SEK 1644) (Statistics Sweden, http://www.scb.se).

### Average net contribution to society

The effect of reduced mortality is determined by the position in the person’s lifecycle. Before the age of 65 years the net contribution to society (production minus consumption) is positive and is negative thereafter. Estimates of the net values were taken from Ekman [27], using the best age-match.

### Cost-effectiveness outcomes

Outcomes, for men and women respectively, are presented as the weighted sum of all subgroup incremental costs, life-years gained (LYG), quality-adjusted life year (QALY) gained, and incremental cost per QALY gained, denoted as incremental cost-utility ratio (ICUR). The weights are the relative population-share of each of the 32 groups taken from the KIMS population used in this study. For comparative reasons, results both excluding (base case) and including indirect costs are presented. In the analysis, both costs and effects were discounted at a 3% rate.

### Sensitivity analyses

All data are subject to uncertainties, either as a consequence of measurement errors or as a result of stochastic variation (variation around the mean) of the underlying variables. In order to assess the extent to which the results were sensitive to the various assumptions, the ICUR was re-estimated for a 1000 set of input values drawn from a probability distribution. The choice of variables that were allowed to vary was decided by a deterministic pre-analysis that identified the variables to which the results were most sensitive. Both univariate and bivariate, non-stochastic, as well as stochastic, sensitivity analyses were performed. The non-stochastic sensitivity analyses examined ranges of specific variables, e.g. intervention costs and first-year treatment effects, in which the ICUR falls below a specified threshold value. Based on the constructed simulation model, Monte Carlo simulations of the incremental cost-utility ratio were performed, using information concerning the distribution and its characteristics for the key parameters entered in the calculation of the ICUR. The stochastic sensitivity analysis included Monte Carlo simulations regarding five variables (variation in subgroups is analysed separately): (1) QoL-AGDHA scores at baseline (assumption: normal distribution); (2) intervention cost (drug utilisation) (assumption normal distribution); (3) yearly mortality risks (assumption: beta distribution); (4) morbidity-related healthcare costs (assumption: log-normal distribution); and (5) first-year treatment effect (assumption: normal distribution). In each case, the point-values used in the deterministic calculations - see Tables 1 and 2 - were used as mean values for the probability distribution. The standard deviations were assumed to be 0.2 times the corresponding mean value for all variables. In addition, we performed simulations assuming a considerably higher relative standard deviation of mortality risks (0.005 for all groups). The method for making models probabilistic follows Briggs *et al*. [28] (see Additional file 1).

#### Short list of modelling assumptions

The following assumption were made in the calculations:

(1) populations: untreated patients during the period 1987–1992 were assumed to be identical to the treated patients from the period 1995–2011 in all respects (but GH treatment);

(2) time-paths: all patients were assumed to fulfil the Markov-assumption in that the likelihood of future events depends only on current state;

(3) co-morbidities: cardio- and cerebrovascular diseases were assumed to capture the extent to which GHD patients suffer from co-morbidities related to their GHD;

(4) multiple health states: it was assumed that there is no transition between co-morbidity states.

(5) mortality risks: assumed to differ between age and gender groups, but to be equal across QoL-AGDHA states;

(6) treatment effects: assumed to occur during the first year. Change in QoL-AGDHA in subsequent years are due to ageing.

(7) indirect costs: It was assumed that sickness-absenteeism is equal between Qol-AGDHA states.

## Results

The results of the simulation model showed a reduction in mortality and an improvement in QoL associated with GHT as compared with no treatment. The mean incremental LYG for patients who received GHT compared with no treatment was 2.3 and 2.1 for men and women, respectively. The average gain in QALY with GHT was 2.3 and 2.3 in men and women, respectively (Table 3).

**Table 3:** **Growth hormone treatment versus no treatment**: **outcomes over a 20**-**year period** (**weighted averages**)

**Outcomes**	**Men**	**Women**
Life-years gained	2.3	2.1
Incremental QALY	2.3	2.3
Incremental costs, €, including direct costs^a^	31,872	41,096
Incremental costs, €, including direct and indirect costs^a,b^	24,607	28,570
Incremental cost per QALY, € (excludes indirect costs^b^)	15,975	20,241
Incremental cost per QALY, € (includes indirect costs^b^)	11,173	10,753
Incremental cost per QALY, € (excludes all indirect effects)	48,252	46,601

^a^Direct costs = treatment cost minus avoided healthcare costs.

^b^Indirect costs = cost of sickness-related absenteeism + lost value of production (production minus consumption due to increased mortality). GHD = growth hormone deficiency; QALY = quality-adjusted life years.

The per-patient total weighted sum of all incremental net costs (treatment cost minus avoided healthcare costs) for GHT compared with no treatment was €31,872 and €41,096 for men and women, respectively. Including indirect costs, the corresponding figures were €24,607 and €28,570. For GHT, drug costs were offset by lower direct healthcare costs (due to fewer CHD and stroke events) and lower indirect costs due to less sickness-related absenteeism. Indirect costs resulting from reduced mortality increased the net cost of GHT due to an on-average negative net contribution to society (value of production minus value of consumption) among the additional survivors. The net per-patient cost (cost of GHD treatment minus changes in cost of general healthcare) increased for both men and women when the costs from reduced sickness-related absenteeism and mortality were taken into account.

The estimated incremental costs per QALY were €15,975 (men) and €20,241 (women) when including only intervention and healthcare costs. Including all indirect costs, the incremental costs per QALY gained were lower; €11,173 and €10,753 for men and women, respectively.

**Figure 2:**
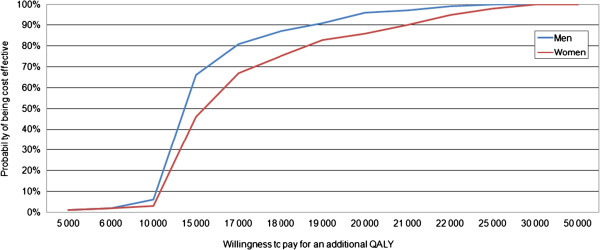
**Cost-effectiveness acceptability curves for men and women.** Each curve indicates, for a specific willingness-to-pay for an additional QLAY (horisontal axis), the likelihood (vertical axis) that GH treatment is cost-effective, compared to no treatment.

**Figure 3:**
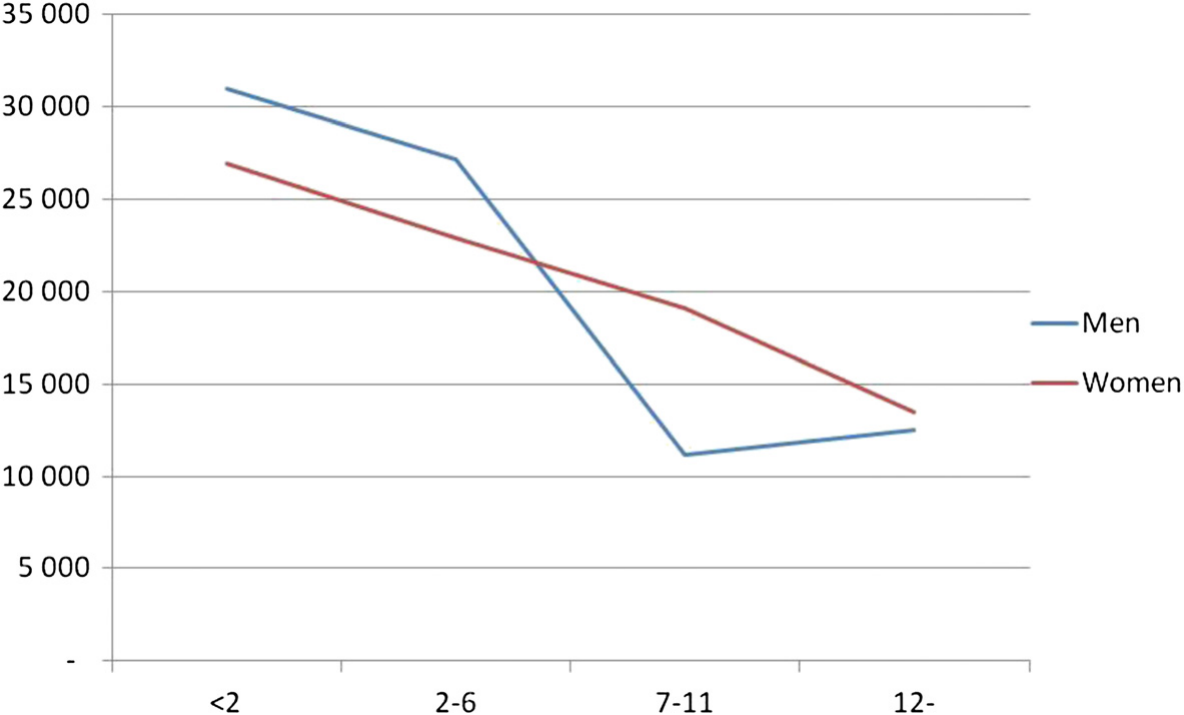
**Cost**-**effectiveness ratios per QOL-AGDHA group and men and women.** Each graph shows the cost-effectiveness ratio (vertical axis) as a function of the QOL-AGDHA score (horisontal axis).

The probabilistic sensitivity cost-effectiveness acceptability curves (Figure 2) suggest that the GH-treatment has a 100% probability of being cost-effective for both men and women at the €55,371 (SEK 500,000) per QALY threshold, which is the threshold level considered to be moderate for society’s willingness to pay for an additional QALY in Sweden [29]. Moreover, the estimated cost-effectiveness ratio varied among the different subgroups. This is illustrated in Figure 3. No significant changes in the results were obtained for the alternative mortality-risk specification.

Non-stochastic sensitivity analysis showed results were most sensitive to changes in intervention costs and first-year treatment effect. Reasonable variations in these variables did not produce an ICUR above the threshold value €55,371 (SEK 500,000) for men and women. Similarly, the stochastic sensitivity analysis produced no value of the cost-utility ratio above €55,371.

## Discussion

In this study, a simulation-model–based cost-effectiveness analysis was conducted comparing GHT with no GHT in adults with GHD in Sweden. The estimated incremental cost-effectiveness ratios ranged between €10,753 to €20,241, which is below the threshold level of €55,371 (SEK 500,000) considered moderate for society’s willingness to pay for an additional QALY in Sweden [29]. Compared with thresholds inferred from the literature, this study’s estimates are substantially lower than those found in Kenkel [30] who reported a willingness to pay for a QALY in the range $74,000 to $450,000, and within the range reported by King *et al*. [31] of $12,500 and $32,200 (2011:1 USD = 0.72 €). Our estimated incremental cost per additional QALY gained suggests that GHT is cost-effective.

Previously, only two studies had been conducted to evaluate the cost-effectiveness of GHT [18,19]. The most recent assessment by Bansback *et al*. [19], part of the NICE Technology Appraisal [32], estimated the incremental cost per QALY gained to be about £50,000, pertaining to the year 2002 in England and Wales. The methodology used in our study differed from that used by Bansback *et al*. with respect to: (1) the incorporation of mortality; (2) the incorporation of indirect effects working through sickness-related absenteeism and the values of productivity and consumption in the economy (arising from mortality); and (3) the method for inferring QALY weights from treatment effects. Moreover, the analysis of our study employed a Markov-type cohort model using transition probabilities from a Swedish population, while Bansback *et al*. simulated morbidity and mortality events in a population using the Framingham risk equations [33], which link cardiovascular events to individual characteristics that, in turn, are influenced by GHT. However, the Framingham risk equations have not been validated for GHD.

The main explanation for our estimates being considerably lower than those published by Bansback *et al*. [19] seems to be indirect effects (mortality effects and work absenteeism). Mortality had virtually no effect on the estimated cost-effectiveness ratios in Bansback *et al*., implying very small differences in mortality risk between treated and untreated patients. Excluding both mortality and work-absenteeism effects in our model increased the cost-effectiveness ratios to about €48,252 and €46,601 for men and women, respectively; hence, in the same range as the estimates obtained by Bansback *et al*. However, the literature suggests that there are significant effects of GHT on mortality [15], which is supported by the data in our model. It is noteworthy, however, that there was a dramatic change in lipid-lowering treatment regimens in the early 1990s in Sweden, which could be considered as a potential confounder. We can speculate that the control cohort most likely did not receive statins, which resulted in higher cardiovascular risks than if they had been prescribed appropriate lipid-lowering treatment. In the general KIMS population, lipid-lowering drugs are reported in approximately 18% of the patients. Therefore, we would not consider the introduction of statins as a confounder. The influence on cost-effectiveness of changes in the values of production and consumption produced by mortality and sickness-related absenteeism was relatively small.

Both this study and Bansback *et al*. [19] employed GHD-specific QoL scores (QoL-AGDHA) in order to calculate utility weights used for obtaining QALYs. While Bansback *et al*. achieved this through an indirect route using three different QoL questionnaires (QoL-AGHDA, Nottingham Health Profile, and SF-36), we utilised published information regarding the link between QoL-AGHDA and EQ-5D in order to calculate utility weights directly [24].

GHT drug doses used in our calculations distinguished between both age and QoL-AGHDA score, while Bansback *et al*. [19] only accounted for differences between age groups. Drug costs are also likely to be lower in our calculations since dosing has declined over time, something that may also explain the difference in cost-effectiveness found between the various studies.

The reduction in the number of sick days is somewhat uncertain as we used information for subjects receiving no treatment compared with the normal population, not for those treated with growth hormone. However, the reduction is similar to the findings in Svensson *et al*. [17] and KIMS data from Sweden further indicate that the number of sick days is low and comparable to the general population for those treated with growth hormones (data on file). Early retirement was not included in the simulation model, since no comparable information was available regarding the effect of GHD and GHT on the risk of being retired at an earlier age. The effect of early retirement on the cost-effectiveness of GHT is likely to be considerable, as the cost imposed on society by early retirement due to GHD has shown to be substantially higher than the corresponding cost for sickness-related absenteeism [6].

An important limitation of this study was that the data used for the simulations were gathered from different sources. It would be ideal to gather all information from the same source, but a randomised controlled trial would not be possible in practice due to ethical considerations. The reliability of the estimates in the study concerning mortality, treatment effects and treatment doses could be disputed. With regard to mortality risks, the data in this study are likely to reflect true differences between GHT and untreated individuals suffering from GHD. The information concerning mortality for untreated individuals comes from the Swedish causes of death registry covering all GHD cases, while the corresponding information regarding GHT individuals comes from a source covering the majority of GHD cases in Sweden (KIMS comprise 95% of the patients treated with Genotropin®; the market share for Genotropin® during the study period was >70%). We have assumed that the clinical characteristics, particularly the severity of hypopituitarism expressed as the number of pituitary hormone deficiencies, were comparable between the cohorts. However, we cannot rule out that the continuous improvements in other pituitary hormonal replacement regimens may have additionally contributed to better results in the KIMS cohort. Furthermore, the data comprising untreated and treated groups, respectively, reflect different time periods (1987–1992 vs 1995–2007). The time difference is likely to be too small for significant differences regarding mortality risks. Moreover, as growth hormone replacement therapy in adults was not authorised in Sweden until 1994, it is reasonable to assume that the vast majority of patients considered untreated did not receive GHT. An additional potential limitation is that the cost-effectiveness results are country specific. However, the constructed model used for the economic evaluation is general and can easily be applied to other countries as long as relevant input data are employed.

Recent evidence have demonstrated yet an additional cause of GHD - traumatic brain injury (TBI), see, for instance, the recent study by Tanriverdi et al [34]. The proportion of TBI that caused GHD is low, however (1.8% of the total KIMS dataset in 2003; [35]). Efforts should be made in future studies to perform separate cost-effectiveness calculations for different patient groups.

Growth hormone was approved in Sweden for treatment in adults with pronounced growth hormone deficiency in 1994, and, hence, our untreated patient group by necessity consisted of those with GHD prior to 1994. The best source of information for this group is the national registries. The majority of the patients included in our untreated group have been diagnosed with pituitary macroadenoma and undergone surgery [36]. GH deficiency appears in about 95% of those patients [36]. The limitations of this approach are (1) that no precise information about the cause(−s) of GHD in the untreated group is available, and (2) that different time periods were compared (untreated 1987–1992, and treated 1995–2011). It may be that being untreated comes with less severe health consequences during the latter period. It cannot be neglected that progress in overall medical treatment and improvement in clinical care may play a certain role in patients’ survival.

In the treated group, the number of patients with isolated GHD was small (n = 34; 7.8%). Thus, the size of the treated group with isolated GHD and the lack of clinical information about hypopituitarism in the comparator group (untreated) makes subgroup analysis impossible. Almost 40% of patients (n = 171) had 1 or 2 additional to GH pituitary hormone deficits and 53% of patients (n = 229) had panhypopituiatrism. With regard to specific deficits 78% of patients had FSH/LH deficiency, 73% - TSH deficiency, 64% - ACTH and 14% - vasopressin. The majority of patients received hormonal replacement as appropriate. Information about hypopituitarism in the comparator group (untreated patients) was not recorded in the hospital discharge registry. Thus, we could not compare treated and untreated cohorts in this respect. However, comparing the KIMS cohort with contemporary patients not in the KIMS cohort demonstrates that the distribution of additional pituitary deficits in the KIMS cohort resembles the overall distribution in patients with hypopituitarism.

## Conclusion

With global healthcare costs escalating, economic evaluations are increasingly used to guide decision-making regarding the funding of new and available drugs. This study fills a gap in the cost-effectiveness literature for the use of GHT in adults with GHD, and suggests that it is a cost-effective therapy in Sweden when considering morbidity and mortality associated with GHD and the impact on QoL.

## Abbreviations

GHT: Growth hormone treatment; GHD: Growth hormone deficiency; QoL: Quality of life; KIMS: Pfizer International Metabolic Database; NFPA: Non functioning pituitary adenoma; QoL-AGHDA: Quality of life assessment of growth hormone deficiency in adults; EQ5D: EuroQoL 5 dimension; CHD: Coronary heart disease; ICUR: Incremental cost utility ratio; QALY: Quality adjusted life year.

## Competing interests

Kristian Bolin was a paid consultant to Pfizer in connection with the development of the economic model and this manuscript. Björn Jonsson was a paid consultant to Pfizer in connection with gathering the data and running the statistical analysis used in the economic model, and to co-author. Rickard Sandin, Maria Koltowska-Häggström, Jane Loftus and Christin Prütz are/were employees of Pfizer and earn Pfizer stock shares. At the time the paper was written, Christin Prütz was an employee of Pfizer AB.

## Authors’ contributions

KB and BJ helped design the study performed the analyses and the modelling. KB helped to draft the manuscript and finish it. MK-H, RS, CP, and JL helped design the study and draft the manuscript. All authors revised, read and approved the final manuscript.

## Supplementary Material

Additional file 1 Supplementary information on modelling, data, and calculations. Click here for file
