# Supplementary material for: The cost-effectiveness of growth hormone replacement therapy (Genotropin®) in hypopituitary adults in Sweden
Source: Cost Eff Resour Alloc. 2013 Sep 30;11:24. doi: 10.1186/1478-7547-11-24 (PMC3850881; doi:10.1186/1478-7547-11-24)
Supplement: Additional file 1 — Supplementary information on modelling, data, and calculations. [file 1478-7547-11-24-S1.doc]

Additional file 1

| **Simulation model: discrete difference equation**  This equation is used for calculating for each year the distribution of the population over the states of the model. The transitions from one state to another are represented by rates of change, and the proportions, which transit from one state to another, are calculated, given age-specific morbidity and mortality rates. The number of individuals in each state at time $t+1$, $N_{i}^{t+1}$ ($i=1,...,32.$), can be expressed by the following discrete difference equation: $N_{i}^{t+1}=N_{i}^{t}+N_{j\to i}^{t}-N_{j\leftarrow i}^{t}$,  where $N_{j\to i}^{t+1}$ is the number of individuals who move to state *i* and $N_{j\leftarrow i}^{t}$ is the number of individuals who leave state *i.* The model performs simultaneous but separate calculations for a population that received GHT and a population that did not receive GHT. |
| --- |
| **Algorithm for translating the QoL-AGHDA score into EQ-5D scores (24)**  This algorithm, recently derived for the Swedish population, was used to calculate year-by-year consecutive utility scores for each subgroup over a time period of 20 years. $U_{ij}(t)=1.05-0.0189\cdot\text{QoL-AGHDA-score}(t)_{\text{ij}}-0.00238\cdot\text{age}_{\text{j}}(t)-0.0127\cdot\text{sex}$ where *i* (age groups) = 18–30, 31–54, 55–65, or 66+ years (mean age in each group has been used, assuming a uniform distribution of individuals; QoL-AGHDA-score for each *t* was calculated based on mean score for each group at baseline); and *j* equals (QoL-AGHDA groups) = score <2, 2–6, 7–11, and 12+. The division of QoL-AGHDA subgroups was based on quartiles. |
| **Sensitivity analysis**  The QoL-AGHDA baseline score and the utilisation of Genotropin®, respectively, were assumed to be normally distributed, while morbidity-related healthcare costs were assumed to be log-normally distributed. In each case, it was assumed that the expected value of the distribution was equal to the observed mean in each group, and that its standard deviation was equal to 20 % of the observed mean. A beta-distribution was assumed regarding mortality risks. In all sensitivity analyses the input variables used in the simulations were varied separately. |
| **Calculation of ICUR estimate** $\sum(\frac{\text{Treatment cost}_{i}-\text{Avoided costs}_{i}}{QALY_{i}})\cdot weight_{i}$ **, which is equal to $\sum(\frac{\text{Treatment} \text{cost}_{\text{i}}\cdot\text{weight}_{i}-\text{Avoided} \text{costs}_{\text{i}}\cdot\text{weight}_{i}}{QALY_{i}})$, but not equal to**  $\frac{\sum\text{Treatment cost}_{\text{i}}\cdot\text{weight}_{i}-\sum\text{Avoided cost}_{\text{i}}\cdot\text{weight}_{i}}{\sum\text{QALY}_{\text{i}}\cdot\text{weight}_{i}}$**.**  **The weight is defined as the number of persons in each subgroup i divided by the total number of persons in the KIMS population.** |
